# Medical Colleges

**Published:** 1854-02

**Authors:** 


The following plain spoken and truthful article form the pen of Professor Charles A. Lee, is a response to one which appeared in this Journal for October. The error of attributing its authorship to our senior, is of small moment, as we believe that nearly the whole medical press of the country would, if called out, unite in condemning the system of government schools.

S. B. H.

*Medical Colleges.*— Our able cotemporary, the *Buffalo Medical Journal*, for November, contains some very judicious remarks on Medical Colleges and Medical Teaching, from the pen of its respected editor, Prof. Flint. We fully agree in the opinion that the complaints against our medical schools are, for the most part, unfounded, and that our present educational system, having free competition for its basis, and resting on the good will of the profession, is the one best adapted to the situation and wants of the country. We have watched the establishment and management of schools depending on government patronage, and we have had opportunities of knowing something of the manner in which medicine is taught in these institutions, and of the position they occupy in public opinion, and we do not hesitate to pronounce them, thus far, decided failures, altogether unsuited to our political institutions, placed as their faculties are above the reach of those motives and stimulants for exertion, which actuate other teachers. We have seen men selected, in such schools, merely from political motives, because they had done good service as party hacks and unscrupulous politicians, to teach branches of science they had never studied, and when elected they have sunk down into a state of very comfortable torpor, under the anaesthetic influence of regular salaries promptly paid, and beyond the reach of all contingency of numbers. We hold that the government system is unsuccessful even in Europe, where social organization and political systems are wholly diverse from our own, but where, if under any circumstances, such a system would be likely to succeed. At Oxford, for example, there are two or three medical professors supported by the State, or by endowment from private bequests, and they rarely have as many as half a dozen students attending their lectures. The cheapness of subjects in Paris, and the great attractions of that city, with its numerous and well managed hospitals, draw together great numbers of students, but the same would happen if the professors were paid by their classes instead of the government. As it is, probably as many students annually assemble at the different schools of Philadelphia, as are to be found in the city Paris. But when it is attempted to establish a government medical school in a small inland country town, away from hospitals, and all opportunities of clinical teaching, paying the professors fixed salaries, whatever may be the number of their students, it requires no great foresight to see that such schools must very imperfectly subserve the objects for which they were established, and that the sooner they are abandoned the better it will be for the interests of medical science. Elected by political influence, the professors hold their offices by the feeble tenure of party supremacy, and when a political change occurs, they are liable to be superseded by another set of political adventurers, who, in their turn, experience the anaesthetic influence of government pap. Besides, the salaries are too small to command men of first-rate talent, for no physician of much eminence would bury himself in a government school, even to gain the title of *Professor*, with an annual income of one thousand dollars only.

We also agree with Prof. Flint in the opinion that the opening of free schools of medicine would have little tendency to improve the character of the profession; and we go still further, and say that nothing would more certainly tend to degrade its character than this. We have now free colleges and others next to free, and what is the character of the graduates who annually go forth from these schools to practice the healing art? Are they not often such as are wholly unfit to make a respectable living in any other calling? Young men too lazy to follow the plow, and too stupid to excel in any other profession, resort to these quack manufactories, where they go through the miserable farce of listening to still more stupid teachers, only to be let loose on the community—a set of ill-mannered, charity empirics, who may be tracked through life by the disgrace which they bring upon an honorable calling. The ranks of quackery, we doubt not, are constantly replenished from such schools as these. To reduce the fees for lectures below a remunerating standard, thus depreciating the value of medical instruction, and inviting into the profession the half-educated, the indolent, those who have failed in other pursuits, who are unable to gain a decent support by any other calling, too stupid for the bar or the pulpit—those who are quacks by nature, and wish the shield of a diploma to cover their charlatanism—this is what some consider the proper mode of elevating the profession, and raising the standard of medical education. We acknowledge that in one point the faculties of these cheap schools are in the right: they seem to have a just estimate of the value of their instruction, and if, instead of half a fee, their teaching was wholly gratuitous, the profession would agree that, if they lacked other acquirements, they had attained a very satisfactory amount of *self-knowledge*. We have a very poor opinion of medical charity scholars, and when we see a young man, willing to accept these proffered advantages, generally delusive, without pay, or at a nominal fee, we look upon him as lacking in self-respect, that sentiment of manly pride and independence which chooses to pay for what is worth paying for, and to work his way honestly by the proceeds of his own labor.

The west as well as the east is becoming flooded with half-educated young men, graduates of Eclectic, Homœopathic, Reformed schools, (so called, which need reforming,) State schools, and half-pay schools, and a majority of them turn out arrant quacks sooner or later, while well educated men of real acquirement and approved skill, are thrust aside by the artifices of these knaves, and the community suffer in consequence. Let free competition prevail in medical teaching, but let it be fair and honorable. Let it be deemed as dishonorable to reduce the fees for lectures below a just remunerating point, as it is for a private practitioner to reduce his charges below the established tariff, and underbid his competitors. Let the States which have entered the race of competition with private enterprise, abandon the field, and adapt their legislation in accordance with the spirit of our institutions, which are hostile to monopolies, and governmental appointments, and leave medical education where it should be left, in the hands of the profession itself. Were it possible to avoid political and partizan influence in making the appointments in State institutions, which it is not, yet the fact that the professors are not dependent on the respect and good will of the profession, but are secure of support, however remiss or negligent in the discharge of their duties, is sufficent to show the impolicy of such a system of medical education, while it is amply confirmed by past experience.— *Ohio Med. & Surg. Jour.*

